# *In Vivo* Functional Analysis of *Drosophila* Robo1 Fibronectin Type-III Repeats

**DOI:** 10.1534/g3.117.300418

**Published:** 2017-12-07

**Authors:** Haley E. Brown, Marie C. Reichert, Timothy A. Evans

**Affiliations:** Department of Biological Sciences, University of Arkansas, Fayetteville, Arkansas 72701

**Keywords:** *Drosophila*, Slit, Robo, axon guidance, midline crossing, fibronectin type-III repeat

## Abstract

The repellant ligand Slit and its Roundabout (Robo) family receptors regulate midline crossing of axons during development of the embryonic central nervous system (CNS). Slit proteins are produced at the midline and signal through Robo receptors to repel axons from the midline. Disruption of Slit-Robo signaling causes ectopic midline-crossing phenotypes in the CNS of a broad range of animals, including insects and vertebrates. While previous studies have investigated the roles of *Drosophila melanogaster* Robo1’s five Immunoglobulin-like (Ig) domains, little is known about the importance of the three evolutionarily conserved Fibronectin (Fn) type-III repeats. We have individually deleted each of *Drosophila* Robo1’s three Fn repeats, and then tested these Robo1 variants *in vitro* to determine their ability to bind Slit in cultured *Drosophila* cells and *in vivo* to investigate the requirement for each domain in regulating Robo1’s embryonic expression pattern, axonal localization, midline repulsive function, and sensitivity to Commissureless (Comm) downregulation. We demonstrate that the Fn repeats are not required for Robo1 to bind Slit or for proper expression of Robo1 in *Drosophila* embryonic neurons. When expressed in a *robo1* mutant background, these variants are able to restore midline repulsion to an extent equivalent to full-length Robo1. We identify a novel requirement for Fn3 in the exclusion of Robo1 from commissures and downregulation of Robo1 by Comm. Our results indicate that each of the *Drosophila* Robo1 Fn repeats are individually dispensable for the protein’s role in midline repulsion, despite the evolutionarily conserved “5 + 3” protein structure.

As the nervous system develops in animal embryos, connections are formed between neurons and other cells via axon guidance. During this process, neurons extend axons through the embryo to form synaptic connections with target cells. In animals with bilateral symmetry, including humans and insects such as the fruit fly *Drosophila melanogaster*, it is critical for each axon to correctly decide whether to remain on its own side of the body or cross the midline to connect with cells on the contralateral side of the body (Evans and Bashaw 2010). Many axons need to cross the midline in order to innervate the opposite side of the body and carry out proper motor functions, necessitating precise temporal regulation of signaling pathways regulating midline attraction and repulsion. Misregulation of midline crossing can lead to a number of neurodevelopmental disorders in humans, including mirror movement synkinesis and horizontal gaze palsy (Izzi and Charron 2011; Nugent *et al.* 2012).

## Slit-Robo signaling in Drosophila

The Slit-Robo pathway is an evolutionarily conserved cellular signaling pathway that regulates midline crossing of axons in the developing CNS in bilaterians, including insects, nematodes, planarians, and vertebrates (Kidd *et al.* 1998a; Zallen *et al.* 1998; Fricke *et al.* 2001; Long *et al.* 2004; Cebrià and Newmark 2007; Cebrià *et al.* 2007; Evans and Bashaw 2012; Yu *et al.* 2014; Li *et al.* 2016a,b). The secreted Slit protein is expressed at the CNS midline and is the canonical ligand for the *Drosophila* Robo family of axon guidance receptors, which signal midline repulsion in response to Slit (Brose *et al.* 1999; Kidd *et al.* 1999). A series of structure/function studies determined that the biochemical interactions between Slit and Robo rely on the leucine-rich repeat (LRR) region of Slit, specifically the LRR2 (D2) domain, binding to the Ig1 and Ig2 domains of Robo receptors (Chen *et al.* 2001; Howitt *et al.* 2004; Liu *et al.* 2004). Further biochemical structure studies suggest that Slit specifically binds to the Ig1 domain of Robo receptors in both insects and mammals (Morlot *et al.* 2007; Fukuhara *et al.* 2008). In wild-type *Drosophila* embryos, Robo1 is expressed at high levels on ipsilateral axons that do not cross the midline, and is nearly undetectable on commissural axons that do cross the midline. In *robo1* mutants, ectopic midline crossing is observed in which FasII-positive longitudinal axons of the medial pathway cross the midline, and commissural axons cross and recross the midline multiple times (Kidd *et al.* 1998b). In *slit* mutants, an even more severe disruption of midline repulsion is observed: axons enter the midline and fail to leave it, collapsing the longitudinal pathways of the axon scaffold (Kidd *et al.* 1999). We have previously reported an *in vivo* structure/function study of *Drosophila* Robo1’s five Ig domains, which confirmed that Ig1 is the only Ig domain essential for Slit binding as well as the receptor’s midline repulsive function in the fly embryonic CNS (Brown *et al.* 2015; Reichert *et al.* 2016).

## Temporal regulation of Robo1 in the developing embryonic CNS

Comm protein is present as a means to negatively regulate Robo1 and allow commissural axons to initially cross the midline once to innervate a target on the contralateral side of the body. Comm expression is transient and functions by endosomal sorting to prevent Robo1 from reaching the growth cone surface (Keleman *et al.* 2002, 2005). When both Comm and Robo1 are present, they are colocalized in vesicles targeted for lysosomal degradation by Comm’s cytoplasmic targeting sequence (Gilestro 2008). The little Robo1 that circumvents this fate and makes it to the plasma membrane is subject to inhibition by Robo2, thus preventing a premature response to Slit (Evans *et al.* 2015). After crossing, *comm* expression is terminated and Robo1 protein is able to accumulate on growth cones to prevent ipsilateral axons from crossing and commissural axons from recrossing the midline inappropriately.

Several factors have been implicated in aiding Robo1 recovery from this strong inhibition. During early embryogenesis, Canoe (Cno) is expressed in ipsilateral axons, while it is later expressed in commissural axons that have crossed the midline once (Slováková *et al.* 2012). This expression pattern, coupled with genetic interaction and *in vitro* experiments, indicates a regulatory role for Cno in which it interacts with Robo1 to enhance the receptor’s localization and midline repulsive function. A recent report indicates that Mummy (Mmy), a gene that encodes the only known *Drosophila* uridine diphosphate-*N*-acetylglucosamine diphosphorylase, maintains the abundance of all three Robo receptors on axons (Manavalan *et al.* 2017).

## Conserved structure of Robo receptors and the roles of Fn domains

The three Roundabout family members in *Drosophila* (Robo1, Robo2, and Robo3) share a conserved 5 + 3 protein structure present in most homologs of the Robo receptor family. This structure consists of five Ig domains, three Fn type-III repeats, a transmembrane domain, and two to four conserved cytoplasmic motifs (CC0, CC1, CC2, and CC3) (Kidd *et al.* 1998a; Bashaw *et al.* 2000). The only known Robo family members to deviate from this characteristic structure are present in the silkworm, *Bombyx mori* (BmRobo1a and BmRobo1b), and in vertebrates (Robo4/Magic Roundabout), where BmRobo1a/b are missing Ig5 and Fn1 and Robo4 is missing Ig3-5 and Fn1 (Huminiecki *et al.* 2002; Li *et al.* 2016a). These homologs serve as a natural means to investigate the functionality of individual domains and suggest that at least some of the Ig and Fn domains are dispensable for the activities of some Robo receptors *in vivo*.

Notably, the mammalian Robo3/Rig-1 receptor does not bind Slit (Zelina *et al.* 2014), but instead interacts with the novel ligand NELL2; this interaction is mediated by one or more of Robo3/Rig-1’s Fn domains (Jaworski *et al.* 2015). Fn type-III repeats have been shown to bind Heparan Sulfate Proteoglycan (HSPG) extracellular matrix proteins (Bencharit *et al.* 2007), which are thought to be important for both Netrin/Frazzled attraction and Slit/Robo repulsion. Although HSPGs have been implicated in regulating both attractive and repulsive signaling at the midline, and heparin has been shown to interact in a ternary complex with Slit and Robo, whether or not heparin/HSPG binding by Fn domains contributes to Slit-Robo signaling *in vivo* is unclear, and our understanding of the role each Fn domain plays in *Drosophila* Robo1’s expression, localization, and midline repulsive function is still lacking (Johnson *et al.* 2004; Fukuhara *et al.* 2008; Ahmed *et al.* 2016).

## An in vivo structure/function analysis of all three Robo1 fibronectin type-III domains

We have previously shown that Ig1 is the only Ig domain of *Drosophila* Robo1 required for the receptor to bind Slit and effectively mediate midline repulsion in the embryonic CNS (Brown *et al.* 2015; Reichert *et al.* 2016). However, despite the conserved structure, none of these domains (Ig1–5) are required for receptor expression, localization, or Comm-dependent downregulation. Are the three Fn repeats likewise dispensable? Here, we address this question by individually deleting the three Fn repeats of Robo1, and examine their ability to bind Slit *in vitro* and characterize *in vivo* receptor expression, localization, and midline repulsive function. We find that none of the three Fn repeats are individually required for the receptor to bind Slit *in vitro* or regulate midline crossing *in vivo*. We also report a unique requirement for Fn3 in the exclusion of Robo1 from commissures and downregulation by Comm.

## Materials and Methods

### Molecular biology

#### Robo1 Fn repeat deletions:

**Figure 1:**
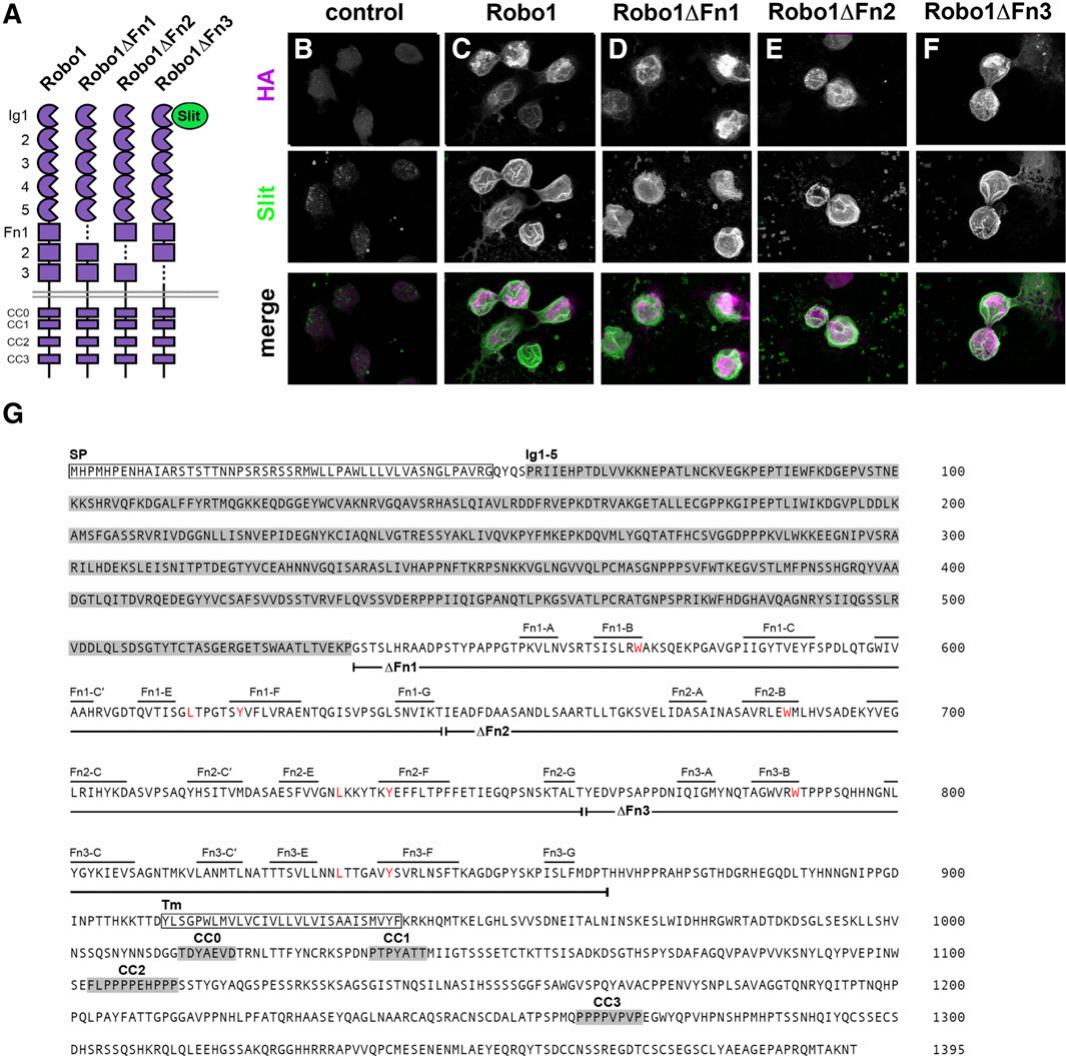
Deletion of individual Fn domains does not interfere with Robo1’s ability to bind Slit in cultured *Drosophila* S2R+ cells. (A) Schematic of the tested Robo1 variants. (B–F) Cells were transfected with HA-tagged Robo1 variants and treated with Slit-conditioned media. After Slit treatment, cells were stained with anti-Slit antibody to detect bound Slit (green) and anti-HA antibody to detect HA-tagged Robo1 variants (magenta). Slit binds only weakly to mock-transfected cells (B), but binds robustly to cells expressing full-length Robo1 (C) or any of the three Fn deletion variants (D–F). (G) Robo1 protein sequence highlighting conserved structural features and illustrating the extent of individual Fn domain deletions. Fn domains have been reannotated based on revised predictions of β-strand locations (annotated above the protein sequence). β-strand nomenclature follows that of Campbell (1994) and Leahy (1996). Amino acids highlighted in red represent a conserved tryptophan residue in strand B, a conserved leucine residue in the E–F loop, and a conserved tyrosine residue in strand F (Leahy 1996). CC, conserved cytoplasmic motif; Fn, fibronectin type-III repeat; Ig1–5, immunoglobulin-like domains 1–5; SP, signal peptide; Tm, transmembrane helix;.

Individual Robo1 Fn repeat deletions were generated via site-directed mutagenesis using Phusion Flash PCR MasterMix (Thermo Scientific), and completely sequenced to ensure that no other mutations were introduced. Robo1 deletion variants include the following amino acid residues, relative to GenBank reference sequence AAF46887: Robo1ΔFn1 (Q52-P534/I646-T1395); Robo1ΔFn2 (Q52-T645/Y763-T1395); and Robo1ΔFn3 (Q52-T762/H866-T1395). Fn domains have been reannotated based on revised predictions of β-strand locations (see Figure 1G).

#### pUAST cloning:

*robo1* coding sequences were cloned as *Bgl*II fragments into p10UASTattB for S2R+ cell transfection. All *robo1* p10UASTattB constructs include identical heterologous 5′ UTR and signal sequences (derived from the *Drosophila wingless* gene) and an N-terminal 3xHA tag.

#### robo1 rescue construct cloning:

Construction of the *robo1* genomic rescue construct was described previously (Brown *et al.* 2015). Full-length and variant Robo1 coding sequences were cloned as *Bgl*II fragments into the *Bam*HI-digested backbone. Robo1 proteins produced from this construct included the endogenous Robo1 signal peptide, and the 4xHA tag was inserted directly upstream of the first Ig domain.

### Genetics

The following *Drosophila* mutant allele was used: *robo1^1^* (also known as *robo^GA285^*). The following *Drosophila* transgenes were used: *P{GAL4-elav.L}3 (elavGAL4)*, *P{10UAS-Comm}86FB* (Reichert *et al.* 2016), *P{robo1*::*HArobo1}* (Brown *et al.* 2015), *P{robo1*::*HArobo1∆Fn1}*, *P{robo1*::*HArobo1∆Fn2}*, and *P{robo1*::*HArobo1∆Fn3}*. Transgenic flies were generated by BestGene, Inc. (Chino Hills, CA) using ΦC31-directed site-specific integration into attP landing sites at cytological position 28E7 (for *robo1* genomic rescue constructs). *robo1* rescue transgenes were introduced onto a *robo1^1^* chromosome via meiotic recombination, and the presence of the *robo1^1^* mutation was confirmed in all recombinant lines by DNA sequencing. All crosses were carried out at 25°.

### Slit binding assay

*Drosophila* S2R+ cells were cultured at 25° in Schneider’s media plus 10% fetal calf serum. To assay Slit binding, cells were plated on poly-l-lysine-coated coverslips in six-well plates (Robo-expressing cells) or 75 cm^2^ cell culture flasks (Slit-expressing cells) at a density of 1–2 × 10^6^ cells/ml, and transfected with pRmHA3-GAL4 (Klueg *et al.* 2002) and HA-tagged p10UAST-Robo, or untagged pUAST-Slit plasmids using Effectene transfection reagent (QIAGEN). GAL4 expression was induced with 0.5 mM CuSO_4_ for 24 hr, then Slit-conditioned media was harvested by adding heparin (2.5 μg/ml) to Slit-transfected cells and incubating at room temperature for 20 min with gentle agitation. Robo-transfected cells were incubated with Slit-conditioned media at room temperature for 20 min, then washed with PBS and fixed for 20 min at 4° in 4% formaldehyde. Cells were permeabilized with PBS + 0.1% Triton X-100, then stained with antibodies diluted in PBS + 2 mg/ml BSA. Antibodies used were: mouse anti-SlitC [#c555.6D, 1:50; Developmental Studies Hybridoma Bank (DSHB)], rabbit anti-HA (#PRB-101C-500, 1:2000; Covance), Cy3-conjugated goat anti-mouse (#115-165-003, 1:500; Jackson Immunoresearch), and Alexa 488-conjugated goat anti-rabbit (#111-545-003, 1:500; Jackson Immunoresearch). After antibody staining, coverslips with cells attached were mounted in Aqua-Poly/Mount (Polysciences, Inc.). Confocal stacks were collected using a Leica SP5 confocal microscope, and processed by Fiji/ImageJ (Schindelin *et al.* 2012) and Adobe Photoshop software.

### Immunofluorescence and imaging

*Drosophila* embryo collection, fixation, and antibody staining were carried out as previously described (Patel 1994). The following antibodies were used: FITC-conjugated goat anti-HRP (#123-095-021, 1:100; Jackson Immunoresearch), Alexa Fluor 488-conjugated goat Anti-HRP (#123-545-021, 1:500; Jackson Immunoresearch), mouse anti-Fasciclin II (#1D4, 1:100; DSHB), mouse anti-βgal (#40-1a, 1:150; DSHB), mouse anti-HA (#MMS-101P-500, 1:1000; Covance), and Cy3-conjugated goat anti-mouse (#115-165-003, 1:1000; Jackson Immunoresearch). Embryos were genotyped using balancer chromosomes carrying *lacZ* markers or by the presence of epitope-tagged transgenes. Nerve cords from embryos of the desired genotype and developmental stage were dissected and mounted in 70% glycerol/PBS. Fluorescent confocal stacks were collected using a Leica SP5 confocal microscope and processed by Fiji/ImageJ (Schindelin *et al.* 2012) and Adobe Photoshop software.

### Data availability

All data generated during this study are included in this published article. Transgenic *Drosophila* lines and recombinant DNA plasmids are available upon request.

## Results

### Robo1 Fn repeats 1–3 are individually dispensable for Slit binding in cultured Drosophila cells

The midline repulsive activity of Robo1 relies on the receptor’s ability to bind its ligand Slit. In our previous investigation of Robo1’s five Ig domains, we determined that Slit binding is essential for midline repulsion and that only the Ig1 domain is required for this process (Brown *et al.* 2015; Reichert *et al.* 2016). Which, if any, of Robo1’s Fn repeats aid Ig1 in Slit binding or midline repulsion? To answer this query, we transfected cultured *Drosophila* SR2+ cells with HA-tagged full-length Robo1 or Robo1 variants missing individual Fn domains, then treated these cells with Slit-expressing media (Figure 1). After Slit treatment, these cells were stained with both anti-HA and anti-Slit to recognize the transgene expressed within the cells and the Slit bound to the cell surface, respectively. All Robo1 Fn variant transgenes (Robo1ΔFn1, Robo1ΔFn2, and Robo1ΔFn3) are able to bind Slit to the same degree as a full-length Robo1 protein and are localized properly to the plasma membrane (Figure 1). Therefore, the Fn repeats are not individually required for Slit binding or membrane localization in cultured cells.

### Robo1 Fn3 is the only domain individually required for exclusion of Robo1 from commissures in vivo

**Figure 2:**
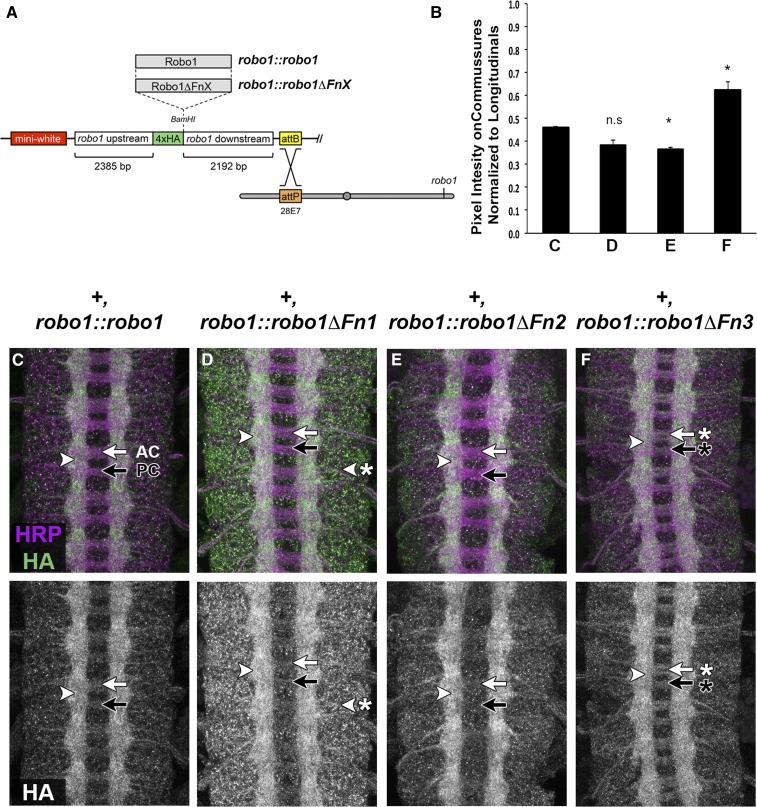
Fn domains 1–3 are not required for axonal localization, and deletion of Fn3 increases Robo1 levels on commissures. (A) Robo1 rescue construct schematic (Brown *et al.*, 2015). HA-tagged *robo1* variant cDNAs are inserted between upstream and downstream flanking sequences, which reproduce *robo1*’*s* endogenous expression pattern. All transgenes are inserted at the same landing site to ensure equivalent expression levels (cytological position 28E7). (B) Average pixel intensity of anti-HA staining on commissural axons normalized to longitudinal axons for the genotypes shown in (C–F). Pixel intensity was measured for commissural axons at five locations per embryo and normalized to pixel intensity of longitudinal axons from the same segment. Normalized commissural expression levels are shown, averaged over three embryos for each genotype. Each variant was compared to +, robo1::robo1 embryos (C) by a Student’s *t*-test, with a Bonferroni correction for multiple comparisons. We detect a statistically significant increase in relative expression levels on commissural axons in embryos expressing Robo1ΔFn3 compared to embryos expressing full-length Robo1 (* *P* < 0.01). (C–F) Stage 16 embryos stained with anti-HA (green) and anti-HRP (magenta) (top), and HA alone (bottom). All transgenic receptors are properly localized on longitudinal axons (arrowhead) and cleared from commissures (arrows), with the exception of Robo1ΔFn3, which is present on commissures (F, arrow with asterisk). Robo1ΔFn1 expression is elevated within cell bodies compared to other transgenes (D, arrowhead with asterisk). AC, anterior commissure; Fn, fibronectin type-III repeat; n.s, not significant; PC, posterior commissure.

To test our Robo1 Fn deletion variants *in vivo*, we utilized a genomic rescue construct in which variant *robo1* cDNAs are cloned into a plasmid containing a regulatory sequence from the endogenous *robo1* gene (Figure 2A) (Spitzweck *et al.* 2010; Brown *et al.* 2015; Reichert *et al.* 2016). These plasmids also contain an attB site to allow ΦC31-directed site-specific integration into attP landing sites at the same cytological location (28E7), to ensure equivalent expression between transgenes.

In wild-type embryos, Robo1 protein is detectable at high levels on longitudinal axons and cleared from commissures. Transgenic HA-tagged Robo1 protein expressed from our rescue construct faithfully reproduces this expression pattern (Figure 2C) (Brown *et al.* 2015; Reichert *et al.* 2016). Each of our Robo1 Fn deletion variants was expressed at similar levels to full-length Robo1 and present on longitudinal axons in embryos carrying the variant transgenes (Figure 2, D–F). However, we noted that Robo1ΔFn3 is not excluded from commissures to the same extent as full-length Robo1 or our other Fn deletion variants (Robo1ΔFn1 and Robo1ΔFn2) (compare commissures in Figure 2, C–F). To quantify this observation, we compared pixel intensities of anti-HA staining for commissural *vs.* longitudinal axons for each of our four transgenes (Figure 2B). We found that HA levels were significantly increased on commissural axons in embryos expressing Robo1ΔFn3 compared to embryos expressing full-length Robo1 (Student’s *t*-test, * *P* < 0.01). These data suggest that Fn3 has a role in preventing Robo1 from reaching the growth cone surface in midline-crossing commissural axons, and/or in maintaining its clearance from commissures after midline crossing.

Additionally, we note that while Robo1ΔFn1 is properly localized to longitudinal axons and cleared from commissures, it displays elevated levels of punctate expression in neuronal cell bodies compared to other Robo1 variants (Figure 2D). We have previously described a similar effect of deleting Robo1’s Ig3 domain (Reichert *et al.* 2016). As with our previously described Robo1 Ig deletion transgenes, we detected no apparent dominant-negative or gain-of-function effects caused by expression of our Robo1 Fn deletion transgenes in otherwise wild-type embryos, even in homozygous embryos carrying two copies of any transgene in addition to two functional copies of the endogenous *robo1* gene.

### Regulation of Robo1 Fn deletion variants by Comm

**Figure 3:**
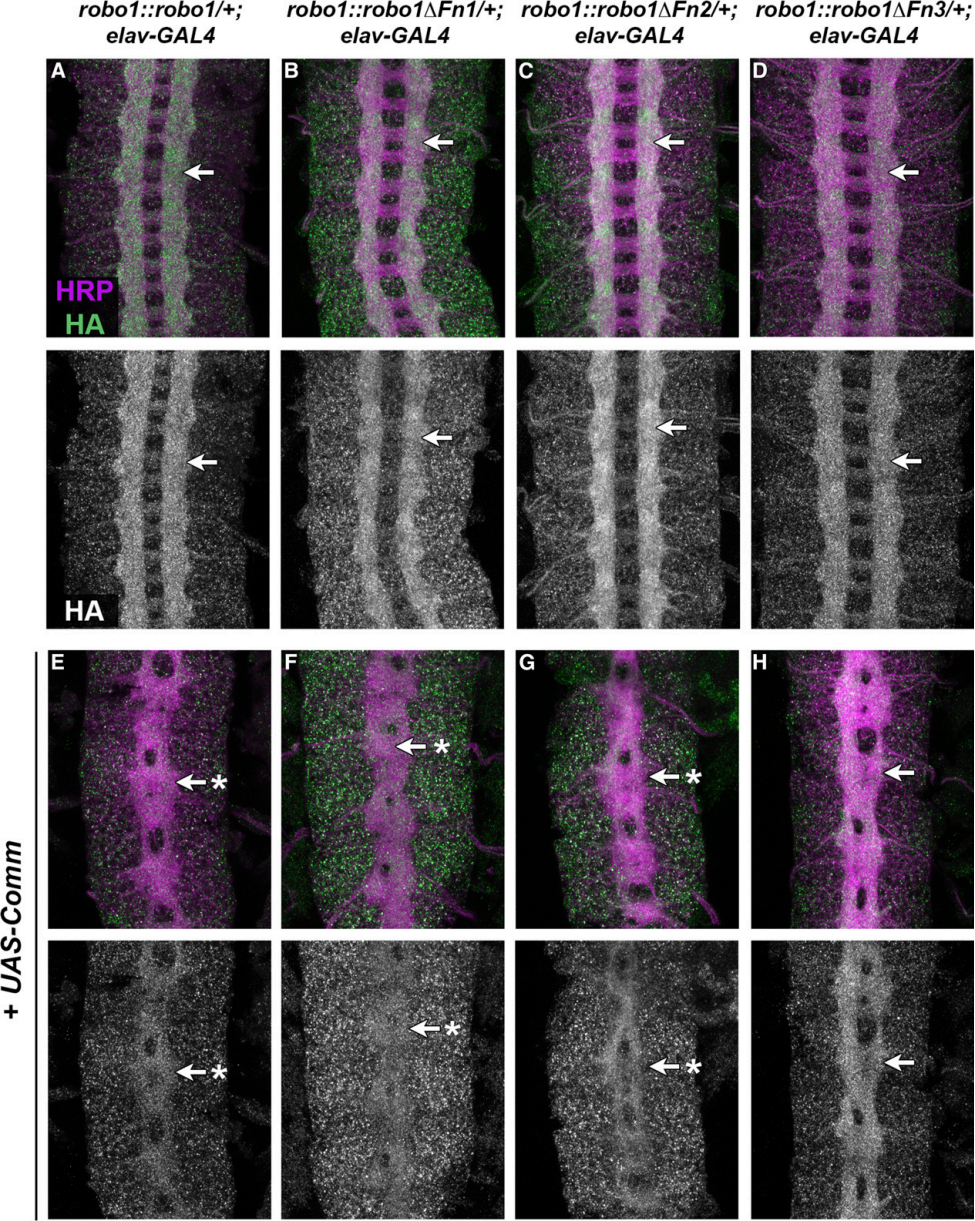
Robo1 Fn1 and Fn2 are not required for regulation by Comm. Stage 16 embryos stained with anti-HA (green) and anti-HRP (magenta). Lower images show HA channel alone of the same embryos. (A–D) Embryos with one copy of the transgene as well as elav-GAL4 display normal Robo1 protein expression among the HA-tagged variants (arrows). (E–G) Homozygous transgenic embryos carrying elav-GAL4 and UAS-Comm show strongly downregulated HA expression among the slit-like collapsed axon scaffold (arrows with asterisks). (H) Robo1ΔFn3 is the only variant that is not downregulated on axons when Comm is misexpressed (arrow). Pairs of sibling embryos shown (A and E; B and F; C and G; and D and H) were stained in the same tube and imaged under the same confocal settings to ensure accurate comparison of HA levels between embryos. Fn, fibronectin type-III repeat.

In *Drosophila*, Comm serves as a negative regulator to the Slit-Robo1 pathway by preventing newly synthesized Robo1 protein from reaching the surface of axonal growth cones. This allows axons to cross the midline and innervate a target on the opposite side of the body (Kidd *et al.* 1998b; Keleman *et al.* 2002, 2005; Gilestro 2008). We have previously reported that none of Robo1’s Ig domains (Ig1–5) are individually required for downregulation by Comm (Brown *et al.* 2015; Reichert *et al.* 2016). To determine whether Robo1’s Fn domains are also individually dispensable for Comm-dependent regulation, we used the GAL4/UAS system to force high levels of ectopic Comm expression in embryos carrying each of our Robo1 Fn deletion variants, and observed the expression and localization of the Robo1 variants within the embryonic nerve cord. Forcing pan-neural Comm expression in embryos encourages a slit-like axon scaffold collapse and the strong downregulation of HA-tagged Robo1 variants on axons (Kidd *et al.* 1998b; Gilestro 2008; Brown *et al.* 2015; Reichert *et al.* 2016). In our transgenic embryos carrying *UAS-Comm* and *elav-GAL4*, expression of each Robo1 variant is strongly reduced compared to embryos carrying *elav-GAL4* alone, with the exception of Robo1ΔFn3 (Figure 3). Here, Robo1ΔFn3 is present on neuronal axons in *UAS-Comm* embryos to the same extent as *elav-GAL4* alone (compare Figure 3, D and H). These results demonstrate that individually deleting Robo1 Fn1 or Fn2 does not disrupt Comm-dependent endosomal sorting, but that Robo1 Fn3 is required for this regulatory process. The strong midline collapse phenotype caused by Comm misexpression in embryos expressing Robo1ΔFn3 suggests that Comm retains the ability to antagonize Robo1ΔFn3 through a nonsorting mechanism, as has previously been described for sorting-deficient forms of Robo1 (Gilestro 2008).

### Robo1’s Fn repeats are not individually required for midline repulsion in vivo

**Figure 4:**
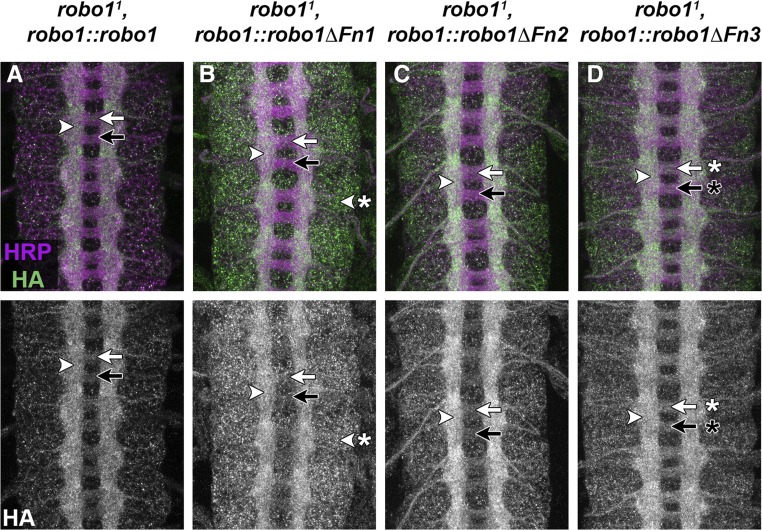
Expression of Robo1 Fn1–3 deletion constructs in robo1 mutant embryos. (A–D) Stage 16 embryos stained with anti-HA (green) and anti-HRP (magenta). Lower images show HA channel alone of the same embryos. Expression of full-length Robo1 in *robo1* mutant embryos is able to fully restore the wild-type axon scaffold and proper receptor localization on axons. (B–D) Each of the Robo1 Fn 1–3 variants shows this wild-type scaffold with HA present on longitudinal axons (arrowheads). As in the wild-type background, Robo1ΔFn1 shows higher protein expression in neuronal cell bodies (B, arrowhead with asterisk) and Robo1ΔFn3 protein is not cleared from commissures (D, arrows with asterisk).

Our previous results established that Robo1 Ig1’s role in Slit binding is paramount to its *in vivo* function in midline repulsion (Brown *et al.* 2015). To determine if Robo1 Fn domains 1–3 also aid in repelling axons from the midline, we introduced our Robo1 variant transgenes into a *robo1* null mutant background and examined their ability to rescue midline repulsion. Restoring expression of any of our Robo1 Fn deletion variants in *robo1* null mutants restored the wild-type appearance of the axon scaffold, as revealed by anti-HRP staining (Figure 4, A–C). Each variant was also properly localized to axons in the absence of endogenous *robo1*. As in a wild-type background, levels of Robo1ΔFn1 were elevated in neuronal cell bodies (Figure 4B), and Robo1ΔFn3 was detectable on both longitudinal and commissural axons (Figure 4D). These results indicate that Robo1ΔFn1, Robo1ΔFn2, and Robo1ΔFn3 are sufficient for normal midline repulsive activity in the absence of endogenous *robo1*.

**Figure 5:**
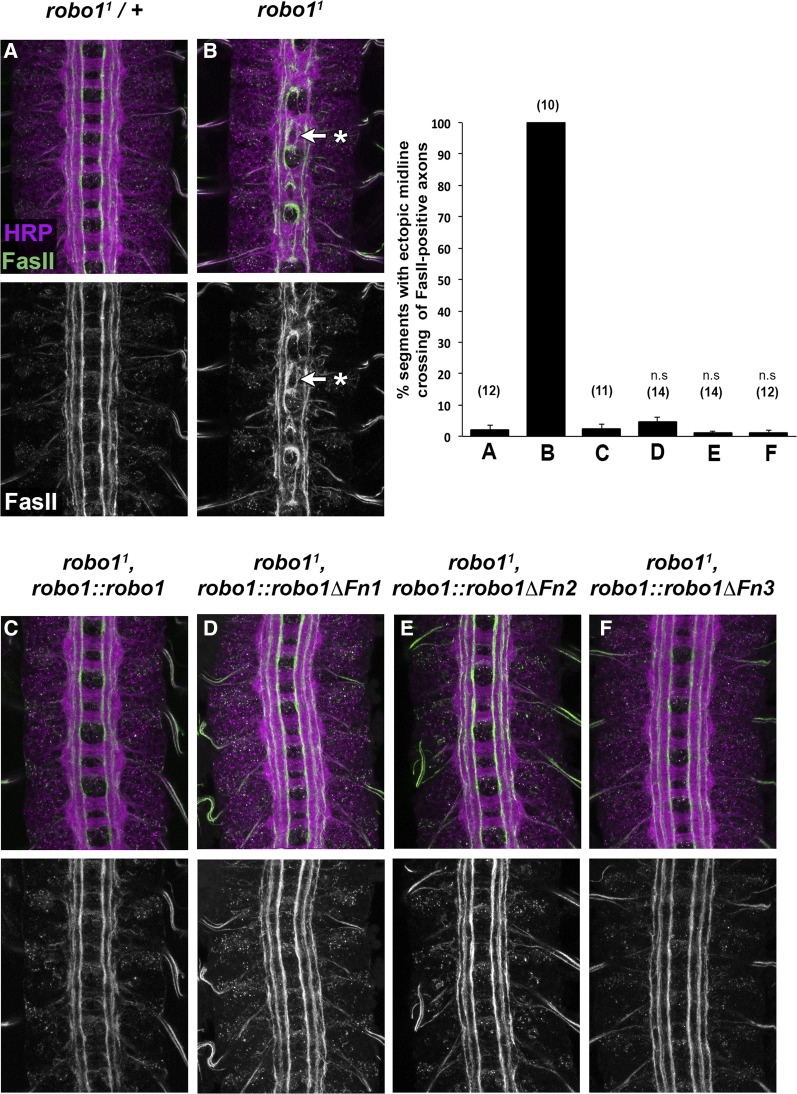
Robo1 Fn1–3 domains are individually dispensable for the receptor’s midline repulsive function. (A–F) Stage 16 embryos stained with anti-FasII (green) and anti-HRP (magenta). Lower images show FasII channel alone of the same embryos. In *robo1* mutant embryos, FasII-positive axons ectopically cross the midline in every segment (B, arrow with asterisk). This phenotype is rescued by a robo1 genomic rescue transgene expressing full-length Robo1 (C) or any of the Fn 1–3 Robo1 deletion variants (D–F). Bar graph shows quantification of ectopic crossing in the genotypes shown (A–F). Error bars indicate SE. Each rescue variant was compared to *robo1^1^*, *robo1*::*robo1* embryos (C) by a Student’s *t*-test, with a Bonferroni correction for multiple comparisons. Number of embryos scored for each genotype is shown in parentheses. n.s, not significant.

To further investigate the ability of our transgenes to rescue midline repulsion in the absence of endogenous *robo1*, we quantified ectopic crossing of FasII-positive axons in stage 16 embryos in each of our rescue backgrounds (Figure 5). In wild-type embryos, the medial, intermediate, and lateral FasII-positive pathways remain distinct on either side of the midline and do not cross. But in *robo1* null mutant embryos, FasII-positive axons ectopically cross and recross the midline in every segment, forming the characteristic roundabouts at the midline for which the receptor was named. By expressing Robo1ΔFn1, Robo1ΔFn2, and Robo1ΔFn3 in a null mutant background, we found that each of these transgenes is able to rescue midline repulsion to the same extent as full-length Robo1 (Figure 5). These results mirror our findings for Robo1 domains Ig2–5, which are each individually dispensable for midline repulsion (Reichert *et al.* 2016), and indicate that the only Robo1 ectodomain element individually necessary for *in vivo* midline repulsion is the Ig1 domain (Brown *et al.* 2015).

## Discussion

In this paper, we have investigated the functional importance of all three Fn type-III repeats of *Drosophila* Robo1. We individually deleted each Fn repeat and examined how the deletion affected Slit binding, receptor expression and localization, commissural clearance, Comm-dependent regulation, and overall midline repulsive function. Our results indicate that Fn3 is the only ectodomain element necessary for commissural clearance and endosomal sorting by Comm, while all three Fn domains are individually dispensable for Slit binding *in vitro* and midline repulsion *in vivo*.

### Evolutionarily conserved Robo1 protein structure

Most members of the Roundabout family have a conserved 5 + 3 protein structure with an ectodomain consisting of five Ig domains and three Fn type-III repeats. The two known exceptions to this characteristic structure are Robo1a/b in the silkworm *B. mori* (which lack Ig5 and Fn1) (Li *et al.* 2016a) and Robo4/Magic Roundabout in vertebrates (which lacks Ig3, Ig4, Ig5, and Fn1) (Huminiecki *et al.* 2002). Together with our previously described Ig deletion variants (Brown *et al.* 2015; Reichert *et al.* 2016), the Fn deletion variants described here reveal that none of these domains are individually required for *Drosophila* Robo1’s role in regulating midline crossing. In fact, we found that, other than Ig1, all of the ectodomain elements are individually dispensable for the receptor’s midline repulsive function. Why then do most Robo1 homologs retain these conserved ectodomain elements? One possibility is that these elements function in a role outside of midline repulsion focused on here. *Drosophila* Robo1 also regulates guidance and targeting of dendrites in the embryo and adult, embryonic muscle migration, embryonic chordotonal sensory neuron migration, and midline crossing of gustatory receptor neurons in the adult fly (Kramer *et al.* 2001; Godenschwege *et al.* 2002; Kraut and Zinn 2004; Dimitrova *et al.* 2008; Mauss *et al.* 2009; Mellert *et al.* 2010). As the *in vivo* mechanisms of these roles are not well understood, perhaps Ig2-Fn3 ectodomain elements of Robo1 aid in these functions by playing either a singular or cooperative role outside of the axon guidance mechanism studied here.

Alternatively, individual ectodomain elements might possess a redundant property that allows one to substitute for another when any individual domain is deleted, or they might collectively serve as spacers to keep Ig1 a certain distance from the plasma membrane to permit Slit binding or facilitate conformational changes required for signaling. Combinatorial deletion studies are underway in our lab to see how many of these domains must be present, or in which combinations, for Robo1 to maintain proper localization, expression, and midline repulsive function.

Robo1 also cooperates with Down syndrome cell adhesion molecule (Dscam1) to promote longitudinal axon guidance in fly embryos in response to a proteolytically processed form of Slit (Alavi *et al.* 2016). We did not observe any consistent or severe defects in longitudinal pathways in any of our rescue backgrounds, suggesting that this activity of Robo1 is also likely to be intact when individual Ig and Fn domains (apart from Ig1) are deleted [this study and Reichert *et al.* (2016)]. However, a more rigorous examination of longitudinal pathways in these backgrounds or in *robo1*, *Dscam1* compound mutants expressing Robo1 Ig and Fn deletion variants may be necessary to rule out a contribution of Ig2–5 and Fn1–3 to this role of Robo1.

### Robo1 Fn3 is required for Comm-dependent endosomal sorting

Comm is a negative regulator of *Drosophila* Robo1, and prevents the receptor from reaching the growth cone surface via colocalization of Comm and Robo1 in lysosomes to be targeted for degradation. Endosomal sorting has been shown to rely on the transmembrane, juxtamembrane, and LPSY sorting motif of Comm and a peri-membrane region of Robo1 spanning 83 amino acids (Gilestro 2008). Using a series of chimeric receptors constructed by swapping various regions of Robo1 and Frazzled, an unrelated receptor that is not sorted by Comm, Gilestro (2008) showed that the peri-membrane region of Robo1 was necessary and sufficient for Comm-dependent sorting in cultured cells, and necessary for Comm sorting *in vivo*. Our results indicate that sequences within the Fn3 domain are also necessary for Comm sorting *in vivo*, and suggest that neither Fn3 nor the peri-membrane region of Robo1 is sufficient for sorting by Comm in embryonic neurons. Notably, both our Robo1ΔFn3 variant and Gilestro’s Robo^SD^ (sorting-defective) variant remain sensitive to antagonism by Comm, as neither variant produces a commissureless phenotype when expressed in place of normal *robo1*, and Comm overexpression is able to mimic a *robo1* loss-of-function phenotype in the presence of either variant.

Finally, we note that while Robo^SD^ is reported to be efficiently cleared from commissural axon segments (Gilestro 2008), our Robo1ΔFn3 variant remains detectable on commissures, suggesting that these two regions of Robo1 (Fn3 and peri-membrane region) may play distinct roles in Comm regulation and/or commissural clearance. Recent evidence suggests that endocytosis of Robo1 may contribute to its downregulation on the surface of midline-crossing growth cones (Chance and Bashaw 2015). Considering the Robo1ΔFn3 construct’s inability to be completely cleared from commissures in wild-type embryos, perhaps the Fn3 domain aids in endocytosis of Robo1, or contains a signal sequence or protein-recognition motif that promotes commissural clearance through another, distinct mechanism, or modulates Robo1’s interaction with other regulatory factors like Cno or Mmy (Slováková *et al.* 2012; Manavalan *et al.* 2017). More experiments will need to be done to investigate these possibilities.

### Conclusions

We have described a functional analysis of all three Fn repeats of *Drosophila* Robo1. This work is the first *in vivo* study of the functional importance of the Fn repeats. We have shown that Fn1–3 are not necessary for Slit binding *in vitro*, nor Robo1’s midline repulsive function *in vivo*. Following our previous studies, we have now individually tested the functionality of each ectodomain element in the Robo1 axon guidance receptor. Together, our results suggest that seven of the eight ectodomain elements in *Drosophila* Robo1 (Ig2–Fn3) are individually dispensable for Slit binding and the receptor’s midline repulsive function.
